# Targeting IL‐17 Presents a Promising Strategy for Treating Diseases Related to the Dysregulation of Lipid Homeostasis

**DOI:** 10.1155/mi/7063696

**Published:** 2026-05-29

**Authors:** Chen Zhang, Yu-yue Xue, Dian-qun Ren, Jia-feng Zhu, Xin Jin, Jie Liu

**Affiliations:** ^1^ Jiaxing Graduate Joint Training Base, Zhejiang Chinese Medical University, Hangzhou, 310053, Zhejiang, China, zcmu.edu.cn; ^2^ Jiaxing Hospital of Traditional Chinese Medicine, Jiaxing, 314000, Zhejiang, China

**Keywords:** atherosclerosis, cholesterol homeostasis, fatty acid synthesis, IL-17, inflammation, lipid metabolism, malignancy, nonalcoholic fatty liver disease, obesity, STAT3 signaling pathway

## Abstract

Lipids are fundamental constituents of cell membranes and play a crucial role in signaling pathways that regulate a wide array of cellular functions. Consequently, the dysregulation of lipid homeostasis is recognized as a significant contributor to metabolic diseases. Given the limitations of current strategies for regulating lipid metabolism, it is essential to explore innovative approaches to modulate lipid homeostasis to effectively address diseases associated with its dysregulation. Inflammation plays a critical role in the regulation of lipid metabolism, acting through various pathways and mechanisms that influence both lipid synthesis and degradation. The involvement of Interleukin‐17 (IL‐17) in inflammatory responses is particularly noteworthy, as recent studies have highlighted its impact on lipid metabolism, particularly in the context of obesity and metabolic disorders. This review critically examines the IL‐17 signaling pathway, elucidates the mechanisms by which IL‐17 affects lipid metabolism, and explores the therapeutic potential of targeting IL‐17 in the treatment of disorders related to lipid homeostasis. This review underscores the promise of targeting IL‐17 signaling pathways as a therapeutic strategy for addressing the dysregulation in lipid homeostasis.

## 1. Introduction

Lipids, including triglycerides, phospholipids, and cholesterol, serve as essential components of cell membranes and are involved in signaling pathways that regulate various cellular functions [1, 2]. The dysregulation of lipid homeostasis is a known contributor to metabolic diseases as it affects the biosynthesis, storage, and degradation of lipids in mammalian cells. This can lead to conditions such as obesity, diabetes, nonalcoholic fatty liver disease (NAFLD), and cardiovascular disease, which are often interlinked through shared metabolic pathways [3–5]. For instance, the risk of cardiovascular disease among individuals who are obese is 2–3 times higher than in those of normal weight [6]. Notably, the prevalence of hyperlipidemia, or dyslipidemia, is indeed a significant public health concern, particularly among adults in China, with a recent study indicating a prevalence rate of ~35.6% [7]. Therefore, employing suitable therapeutic strategies to restore disrupted lipid homeostasis to normal levels is of significant clinical importance.

Managing dyslipidemia effectively involves a combination of pharmacological interventions and lifestyle modifications. Statins are essential for lowering cholesterol by inhibiting HMG‐CoA reductase [8], thereby reducing low‐density lipoprotein cholesterol (LDL‐C) levels and cardiovascular risk [9]. They significantly decrease the risk of atherosclerotic events but can cause muscle issues and increase diabetes risk, requiring vigilant blood glucose monitoring [10]. Fibrates are lipid‐modifying drugs that primarily lower triglycerides and raise high‐density lipoprotein cholesterol (HDL‐C) by activating peroxisome proliferator‐activated receptors (PPARs) involved in lipid metabolism. They are especially beneficial for those with high triglycerides and can be combined with statins for better lipid control [11]. However, fibrates can cause liver and kidney damage, particularly in those with pre‐existing conditions [12] and may not prevent cardiovascular events. Additionally, they can increase muscle‐related side effects when used with statins [13]. Cholesterol absorption inhibitors like ezetimibe block cholesterol absorption in the intestine, reducing LDL‐C levels by decreasing the amount of cholesterol delivered to the liver. Often used with statins for further LDL‐C reduction, especially in patients not reaching target levels with statins alone [14]. However, they don’t affect cholesterol production, may trigger increased synthesis when used alone [15], and benefits vary among patients, highlighting the need for personalized treatment [16]. PCSK9 inhibitors are a new class of drugs that effectively lower cholesterol, especially in patients who can’t tolerate statins or have familial hypercholesterolemia. They work by blocking the PCSK9 protein, which normally breaks down low‐density lipoproteins (LDL) receptors in the liver, allowing the liver to remove more LDL‐C from the blood and significantly reduce its levels [17, 18]. However, their high cost and the need for injections can make them less accessible and harder for patients to stick with [19, 20]. Lifestyle modifications are crucial for dyslipidemia management, including dietary changes, increased physical activity, and maintaining a healthy weight. These can enhance drug effects and promote long‐term cardiovascular health [21]. However, lifestyle changes for managing dyslipidemia face challenges, such as adherence due to significant commitment, lack of motivation, time, or support, leading to suboptimal lipid management and increased cardiovascular risk [22]. Additionally, individual responses vary, and for severe cases, medication is often needed, complicating treatment and increasing costs [23]. Consequently, considering the limitations inherent in existing strategies for regulating lipid metabolism, it is imperative to investigate novel approaches for modulating lipid homeostasis in order to address diseases associated with the dysregulation of lipid homeostasis.

Inflammation is critically involved in the regulation of lipid metabolism, operating through a variety of pathways and mechanisms that affect both lipid synthesis and degradation. A central element of this interaction is the influence of proinflammatory cytokines, which are known to modulate lipid metabolism by promoting lipolysis and inhibiting lipid synthesis. Cytokines such as tumor necrosis factor‐*α* (TNF‐*α*) and interleukins, secreted by adipose tissue and other immune cells, regulate the proliferation and apoptosis of adipocytes, thereby impacting lipid homeostasis [24]. Additionally, nuclear receptors such as PPARs and liver X receptors (LXRs), which are activated by lipid metabolites, play crucial roles as regulators of both lipid metabolism and inflammation. These receptors can modulate the expression of genes involved in metabolic and inflammatory pathways, rendering them promising targets for therapeutic interventions in metabolic diseases [25]. The involvement of Interleukin‐17 (IL‐17) in inflammatory responses is particularly intriguing, as recent studies have demonstrated its impact on lipid metabolism, especially in the context of obesity and metabolic disorders. IL‐17 inhibits adipogenesis by suppressing key transcription factors and regulates genes associated with lipid and glucose metabolism [26, 27]. Furthermore, IL‐17 plays a role in atherosclerosis and hepatic lipid metabolism. Inhibition of IL‐17A in adipocytes has been shown to suppress diet‐induced obesity (DIO) and metabolic disorders in murine models [28]. Additionally, IL‐17 interacts with other cytokines and signaling pathways, such as the IL‐17/IL‐23 axis, influencing lipid metabolism in NAFLD [29]. These findings underscore the potential of targeting IL‐17 signaling pathways as a therapeutic strategy for addressing dysregulation in lipid homeostasis.

This review examines the IL‐17 signaling pathway, elucidates the mechanisms through which IL‐17 influences lipid metabolism, and explores the potential of targeting IL‐17 in the treatment of lipid homeostasis disorders. These insights may contribute to the development of novel therapeutic strategies for metabolic diseases.

### 1.1. IL‐17 Signaling Pathway and Its Activation

The IL‐17 cytokine family, encompassing IL‐17A through IL‐17F, is integral to both innate and adaptive immune responses and is implicated in the pathogenesis of numerous inflammatory and autoimmune disorders. IL‐17A, the most extensively researched member, is recognized for its proinflammatory characteristics and its role in autoimmune diseases such as psoriasis and rheumatoid arthritis. It functions by facilitating the recruitment of neutrophils and stimulating the production of additional proinflammatory cytokines and chemokines, thereby intensifying inflammatory responses [30]. Recent research has broadened the scope of our understanding of the IL‐17 family beyond IL‐17A. For example, IL‐17F, which exhibits functional similarities to IL‐17A, has been associated with the inflammatory pathways involved in conditions such as spondyloarthritis and psoriasis [31]. Furthermore, the IL‐17 cytokine family extends beyond merely proinflammatory functions. IL‐17E, also referred to as IL‐25, participates in allergic reactions and confers protection against parasitic infections. This cytokine’s capacity to modulate immune responses highlights the extensive functional diversity within the IL‐17 family [32]. Additionally, the IL‐17 family encompasses less‐explored members such as IL‐17D, which has been demonstrated to stimulate the production of inflammatory cytokines, although its exact role in disease pathogenesis is still under investigation. Recent studies have proposed potential functions for IL‐17D in tumor biology and infections, suggesting that this cytokine may have more extensive implications in immune regulation than previously understood [33].

**Figure 1:**
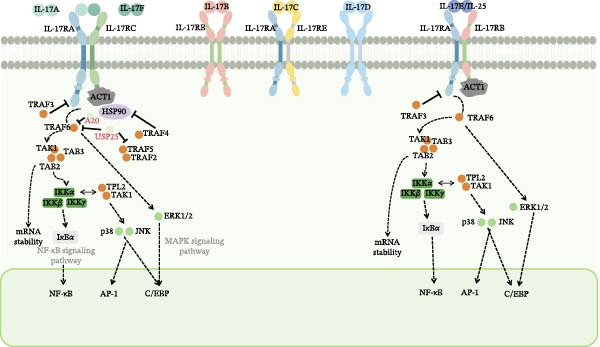
Illustrates the IL‐17 family members, their receptor complexes, and the downstream signaling pathways involved in lipid metabolism. The IL‐17 cytokine family consists of six members (IL‐17A to IL‐17F). Among them, IL‐17A and IL‐17F primarily signal through a heterodimeric receptor complex composed of IL‐17RA and IL‐17RC. Upon ligand binding, the adaptor protein ACT1, which interacts with the SEFIR domain interactions, initiating the activation of downstream signaling cascades, including the NF‐*κ*B, MAPK (ERK, JNK, and p38), and STAT3 pathways. These signaling pathways regulate the transcription of genes involved in fatty acid synthesis, triglyceride metabolism, cholesterol homeostasis, and inflammatory responses. Abbreviations: A20, TNFAIP3 (tumor necrosis factor alpha‐induced protein 3); ACT1, NF‐*κ*B activator 1; AP‐1, activator protein 1; C/EBP, CCAAT/enhancer‐binding protein; ERK, extracellular signal‐regulated kinase; HSP90, heat shock protein 90; IKK, I*κ*B kinase; I*κ*B*α*, inhibitor of NF‐*κ*B alpha; IL‐17, interleukin‐17; IL‐17RA/RC, interleukin‐17 receptor A/C; JNK, c‐Jun N‐terminal kinase; MAPK, mitogen‐activated protein kinase; SEFIR, SEF/IL‐17 receptor domain; TAB1/2/3, TAK1‐binding protein 1/2/3; TAK1, transforming growth factor‐*β*‐activated kinase 1; TPL2, tumor progression locus 2; TRAF, tumor necrosis factor receptor‐associated factor; USP25, ubiquitin‐specific peptidase 25.

**Figure 2:**
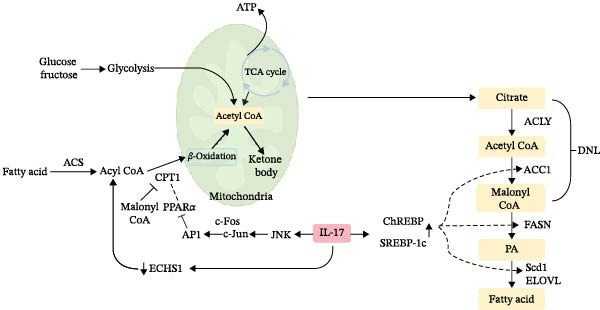
Effects of IL‐17 signaling on fatty acid metabolism. IL‐17 signaling regulates key enzymes involved in de novo lipogenesis and fatty acid *β*‐oxidation. It promotes lipid accumulation by enhancing the expression of lipogenic enzymes such as acetyl‐CoA carboxylase and fatty acid synthase, while suppressing fatty acid catabolism through inhibition of *β*‐oxidation‐related genes. Abbreviations: ACC1, acetyl‐CoA carboxylase 1; ACLY, ATP‐citrate lyase; ACS, acyl‐CoA synthetase; DNL, de novo lipogenesis; ELOVL, elongation of very long‐chain fatty acids; FASN, fatty acid synthase; PA, palmitic acid.

**Figure 3:**
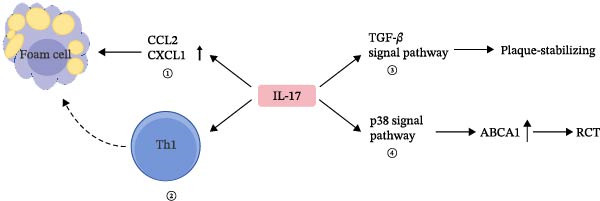
IL‐17 regulates cholesterol metabolism. IL‐17 exerts dual effects on atherosclerosis progression. On the one hand, it promotes inflammation by inducing the secretion of chemokines such as CCL2 and CXCL1 and enhancing Th1 cell activity, thereby increasing macrophage recruitment and foam cell formation. On the other hand, IL‐17 may stabilize atherosclerotic plaques by activating the TGF‐*β* signaling pathway and the p38 MAPK pathway, thereby reducing lipid deposition and the risk of plaque rupture. Abbreviations: ABCA1, ATP‐binding cassette transporter A1; CCL2, C‐C motif chemokine ligand 2; CXCL1, C‐X‐C motif chemokine ligand 1; RCT, reverse cholesterol transport; Th1, T helper 1 cells.

The initiation of the IL‐17 signaling pathway is predominantly facilitated by the interaction of IL‐17 with its receptor complex, comprising the IL‐17RA and IL‐17RC subunits. This interaction instigates a series of intracellular signaling events involving several critical molecules and pathways. A central mechanism of IL‐17 signaling is the activation of the nuclear factor kappa‐light‐chain‐enhancer of activated B cells (NF‐*κ*B) pathway. Following IL‐17 binding, the adaptor protein ACT1, also known as TRAF3IP2, is recruited to the receptor complex. ACT1 subsequently interacts with tumor necrosis factor receptor‐associated factor 6 (TRAF6), culminating in the activation of the NF‐*κ*B pathway. This activation leads to the transcription of proinflammatory cytokines and chemokines, which play an essential role in the immune response [34]. In addition to NF‐*κ*B, the mitogen‐activated protein kinase (MAPK) pathways, encompassing extracellular signal‐regulated kinase (ERK), c‐Jun N‐terminal kinase (JNK), and p38 MAPK, are activated by IL‐17 signaling. These pathways contribute to the expression of inflammatory mediators and participate in various cellular processes, including proliferation and apoptosis [35]. Furthermore, the phosphoinositide 3‐kinase (PI3K)/Akt pathway constitutes a crucial element of IL‐17 signaling. Activation of this pathway by IL‐17 results in the production of proinflammatory cytokines and promotes cell survival and proliferation. Additionally, the PI3K/Akt pathway is implicated in the regulation of other signaling molecules, such as mTOR, which is instrumental in the differentiation of T helper 17 (Th17) cells [36]. Moreover, the signaling of IL‐17 can be influenced by a variety of factors, including other cytokines and environmental stimuli. For example, IL‐6 can potentiate the effects of IL‐17 by activating the signal transducer and activator of transcription 3 (STAT3) pathway, which is essential for the differentiation and function of Th17 cells. This interaction underscores the intricate network of signaling pathways in which IL‐17 is involved, demonstrating its capacity to integrate signals from diverse sources to modulate immune responses [37]. In essence, the activation of the IL‐17 signaling pathway entails a sophisticated interplay of multiple signaling cascades, such as NF‐*κ*B, MAPK, and PI3K/Akt pathways (Figure 1). These pathways collaborate to regulate the expression of inflammatory mediators and contribute to the immune response, underscoring the pivotal role of IL‐17 in both health and disease. The effects of IL‐17 on fatty acid synthesis, triglyceride metabolism, and cholesterol homeostasis are illustrated in Figures 2 and 3.

### 1.2. IL‐17 Negatively Regulates Adipocyte Differentiation and Metabolism

**Figure 4:**
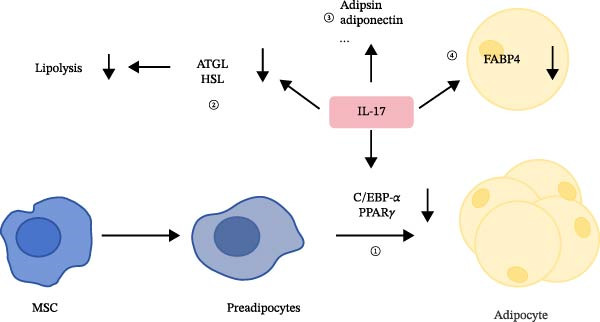
Effects of IL‐17 on adipocyte differentiation and metabolism. IL‐17 inhibits adipocyte differentiation by suppressing transcription factors such as C/EBP‐*α* and PPAR*γ*. It also regulates lipid metabolism by reducing the expression of lipolysis‐related enzymes, including adipose triglyceride lipase, hormone‐sensitive lipase, and adipsin. In addition, IL‐17 decreases fatty acid‐binding protein 4 expression, thereby reducing lipid accumulation in adipocytes. Abbreviations: ATGL, adipose triglyceride lipase; FABP4, fatty acid‐binding protein 4; HSL, hormone‐sensitive lipase; MSC, mesenchymal stem cell.

IL‐17 has been demonstrated to impede the differentiation of mesenchymal stem cells (MSCs) into human adipocytes [38–40]. In mice deficient in IL‐17, there is a significant upregulation in the mRNA levels of proadipogenic transcription factors, including CCAAT/enhancer‐binding protein alpha (C/EBP‐*α*) and peroxisome proliferator‐activated receptor gamma (PPARγ), as well as hormone‐sensitive lipase (HSL), a crucial enzyme in lipolysis [41]. This observation implies that IL‐17 normally suppresses the expression of these genes, thereby restricting the differentiation of preadipocytes into mature adipocytes under physiological conditions [27]. Furthermore, IL‐17 has been shown to decrease the expression of fatty acid‐binding protein 4 (FABP4) in adipocytes, which is essential for fatty acid uptake and storage, as depicted in Figure 4. The downregulation of FABP4 contributes to the inhibition of lipid accumulation within cells [42]. Additionally, IL‐17 exerts an inhibitory influence on molecules involved in lipid droplet catabolism and adipocyte function, such as adipose triglyceride lipase (ATGL) and adipsin, as shown in Figure 4, thereby further impacting the energy mobilization and insulin sensitivity of adipocytes [43].

IL‐17 not only negatively regulates preadipocyte differentiation but also induces functional abnormalities in mature adipocytes by impairing lipid and glucose uptake [44]. Additionally, IL‐17 exacerbates insulin resistance in adipose tissues. IL‐17A can phosphorylate and modify PPARγ through a cyclin‐dependent kinase 5 (CDK5)‐dependent pathway, thereby altering the expression of downstream protective genes such as adiponectin and adipsin [28]. This modification results in the pathological reprogramming of adipocytes, which promotes DIO and metabolic syndrome.

IL‐17 plays a bifunctional role in the regulation of adipose tissue homeostasis. Under normal physiological conditions, IL‐17 inhibits excessive adipogenesis; however, in the context of inflammation or elevated IL‐17 levels, it may compromise the functionality of mature adipocytes. This impairment is implicated in the pathogenesis of obesity and metabolic syndrome, both of which have emerged as significant public health challenges globally [45]. Obesity is frequently associated with metabolic abnormalities, including insulin resistance, hyperglycemia, and hyperlipidemia, which can predispose individuals to cardiovascular diseases, diabetes, and other severe health complications. The onset of obesity is intricately connected to the excessive proliferation of adipocytes and disruptions in lipid storage. It is not merely characterized by fat accumulation but represents a complex pathological process involving immune, metabolic, and endocrine dysregulation. Adipose tissue possesses endocrine functions, secreting various hormones and cytokines that are pivotal in the progression of obesity and metabolic disorders. In the context of obesity, characterized by a chronic inflammatory state, the activation of immune cells and the subsequent release of cytokines are pivotal in driving pathological alterations [46]. Baicalein ameliorates lipid metabolism disorders associated with obesity by inhibiting the IL‐17 signaling pathway [47]. Consequently, the IL‐17 signaling pathway emerges as a promising therapeutic target for modulating adipocytes and addressing their related metabolic dysfunctions.

### 1.3. Targeting IL‐17 Represents a Promising Therapeutic Strategy for Addressing Diseases Associated With the Dysregulation of Fatty Acid Metabolism

Fatty acid metabolism is integral to various physiological processes and is intricately associated with numerous diseases. This metabolic process encompasses several pathways, including *β*‐oxidation, synthesis, desaturation, elongation, and peroxidation, all of which are essential for maintaining energy homeostasis and cellular function. Dysregulation within these pathways can result in a spectrum of metabolic disorders and diseases. Emerging evidence indicates that IL‐17 plays a pivotal role in modulating fatty acid metabolism. IL‐17 impedes fatty acid *β*‐oxidation through multiple signaling pathways. Specifically, IL‐17 activates the c‐Jun N‐terminal kinase (JNK) pathway (Figure 2), thereby suppressing the expression of PPAR*α* and its downstream target genes, including carnitine palmitoyltransferase 1A (CPT1a), medium‐chain acyl‐CoA dehydrogenase (MCAD), and acyl‐CoA oxidase 1 (ACOX1). This suppression limits the mitochondrial uptake and catabolism of fatty acids [48, 49]. Additionally, IL‐17 downregulates enoyl‐CoA hydratase short chain 1 (ECHS1) expression (Figure 2), disrupting the second step of the *β*‐oxidation hydration reaction, leading to the accumulation of acyl‐CoA and further exacerbating lipid accumulation [50, 51]. Animal studies have demonstrated that the administration of exogenous IL‐17A significantly inhibited the expression of *β*‐oxidation genes in a mouse model induced by a high‐fat diet, resulting in increased triacylglycerol (TG) accumulation in hepatic tissue [52].

The circadian rhythm of IL‐17 production by γδT cells plays a pivotal role in maintaining adipose tissue homeostasis. Research on γδT cells within adipose tissue has demonstrated that these cells secrete IL‐17A/F in a circadian manner, which subsequently activates the IL‐17 receptor C (IL‐17RC) and enhances the expression of de novo lipogenesis (DNL)‐related genes, such as stearoyl‐CoA desaturase 1 (Scd1) [53]. Nevertheless, excessive activation of this synthetic pathway can result in the overaccumulation of fatty acids and triglycerides in the liver. An additional animal study indicated that IL‐17 upregulates the expression of sterol regulatory element‐binding protein‐1c (SREBP‐1c) and carbohydrate response element‐binding protein (ChREBP) (Figure 2), thereby facilitating fatty acid synthesis and DNL [54]. SREBP‐1c predominantly regulates lipid synthesis in the liver and adipose tissue by directly binding to sterol regulatory elements (SREs) in the promoters of target genes, thereby promoting the expression of key enzymes such as fatty acid synthase (FASN), acetyl‐CoA carboxylase (ACC), and Scd1 [55]. Furthermore, SREBP‐1c represents the initial regulatory step in triglyceride synthesis and serves as a critical transcriptional regulator of de novo adipogenesis [56]. Nevertheless, research has demonstrated that in adipocytes, NF‐*κ*B is capable of directly binding to the promoter region of SREBP‐1c, thus inhibiting its excessive activation [57–59]. ChREBP, a member of the basic helix‐loop‐helix leucine zipper (bHLH‐Zip) family, predominantly governs carbohydrate metabolism within the liver [60]. It regulates the synthesis, elongation, and desaturation of fatty acids by inducing the expression of ACC1 and FASN for synthesis, Elovl6 for elongation, and Scd1 for desaturation [61, 62].

In recent years, NAFLD has become the most prevalent liver disorder globally [63]. NAFLD is primarily characterized by the accumulation of hepatic fat, hepatocellular injury, and inflammation, with potential progression to liver fibrosis, cirrhosis, or hepatocellular carcinoma (HCC) [63, 64]. Disruptions in fatty acid metabolism play a direct role in the onset and progression of NAFLD [65, 66]. A Mendelian randomization study has identified a positive association between IL‐17 and an increased risk of NAFLD [67]. Neutralization of IL‐17 has been shown to protect against NAFLD progression and mitigate liver injury in obese mice subjected to lipopolysaccharide (LPS) challenge [48, 68, 69]. The aberrant activation of SREBP1c and ChREBP enhances hepatic DNL, while the JNK signaling pathway regulates peroxisome proliferator‐activated receptor alpha (PPAR*α*), MCAD, and carnitine palmitoyltransferase 1 (CPT1), as well as nuclear receptor corepressor 1 (Ncor1) to suppress fatty acid *β*‐oxidation, collectively contributing to intrahepatic lipid accumulation and lipid droplet formation [70]. Furthermore, NF‐*κ*B may influence histone deacetylase 1 (HADC1) via its P50 subunit, promoting lipid droplet formation in hepatocytes through the desuccinylation of SREBP1c [59]. Furthermore, IL‐17 contributes to hepatic inflammation by activating immune cells, including Kupffer cells and hepatic stellate cells (HSCs), which leads to the induction of IL‐6 and TNF‐*α* expression and disruption of insulin signaling pathways. This cascade of events exacerbates hepatocellular injury and fibrosis [68, 71, 72]. Treatment with anti‐IL‐17 monoclonal antibodies has been shown to significantly enhance hepatic function, decrease lipid accumulation, and inhibit both Kupffer cell activation and proinflammatory cytokine production. These effects are associated with the suppression of the NF‐*κ*B signaling pathway [70]. Consequently, targeting the IL‐17 signaling pathway has emerged as a promising therapeutic strategy for addressing diseases associated with dysregulated fatty acid metabolism.

### 1.4. Targeting IL‐17 Constitutes a Promising Therapeutic Strategy for Treating Disease Related to the Dysregulation of Cholesterol Metabolism

Cholesterol constitutes an essential element of cellular membranes and is integral to numerous physiological processes, including the synthesis of hormones, bile acids, and vitamin D [73, 74]. The liver serves as the principal site for cholesterol biosynthesis, primarily through the mevalonate pathway [75]. Within this pathway, 3‐hydroxy‐3‐methylglutaryl‐coenzyme A (HMG‐CoA) reductase acts as the rate‐limiting enzyme, thereby playing a critical regulatory role. Cholesterol is transported from the liver to peripheral tissues via LDL and is subsequently returned to the liver through reverse cholesterol transport, a process mediated by high‐density lipoproteins (HDL). This reverse transport mechanism is regulated by ATP‐binding cassette (ABC) transporters, such as ABCA1 and ABCG1 [76].

Accumulating clinical and experimental evidence suggests that IL‐17 serves as a critical link between chronic inflammation and cholesterol dysregulation, particularly in inflammatory conditions such as psoriasis, where there is a high prevalence of metabolic syndrome and cardiovascular comorbidities. Individuals with psoriasis often exhibit decreased levels of high‐density lipoprotein cholesterol (HDL‐C) and increased levels of low‐density lipoprotein cholesterol (LDL‐C) and TG, which reflect a characteristic dyslipidemic profile associated with chronic systemic inflammation [77, 78]. Lipidomic and metabolomic analyses further demonstrate that psoriasis is associated with profound alterations in long‐chain fatty acids and cholesterol‐related lipid species, which are closely correlated with systemic inflammation severity and disease activity [79]. Emerging evidence suggests that IL‐17A signaling directly contributes to lipid metabolic disturbances in psoriasis by modulating cholesterol homeostasis and inflammatory lipid pathways. The activity of IL‐17A during psoriatic inflammation is contingent upon the availability of intracellular cholesterol. Alterations in cholesterol accumulation can exacerbate IL‐17–induced keratinocyte dysfunction and plaque formation, thereby establishing a connection between lipid metabolism and both cutaneous and systemic inflammation [80]. Notably, under psoriatic conditions, IL‐17–producing γδ T cells undergo metabolic reprogramming characterized by ACC1‐dependent DNL, which sustains IL‐17 production and amplifies local and systemic inflammatory responses [81]. In parallel, obesity‐associated dyslipidemia further aggravates IL‐17–driven inflammation as saturated fatty acids promote excessive neutrophil extracellular trap (NET) formation, thereby enhancing cutaneous inflammation and contributing to reduced responsiveness to biologic therapies in obesity‐related psoriasis [82]. The application of IL‐17 monoclonal antibodies in the treatment of psoriasis has become prevalent, often resulting in clinical improvements that are accompanied by a reduction in blood lipid levels [78, 83]. Metabolomic and lipidomic analyses of psoriasis patients undergoing treatment with IL‐17A monoclonal antibodies have demonstrated notable enhancements in serum lipid profiles, including reductions in atherogenic long‐chain saturated fatty acids and normalization of lipid subclasses associated with cardiovascular risk [79]. These lipidomic changes are accompanied by decreased systemic inflammation and may contribute to a reduced cardiovascular risk burden in patients receiving anti‐IL‐17 therapy, highlighting the dual anti‐inflammatory and metabolic advantages of IL‐17 blockade in addressing psoriasis‐associated dyslipidemia.

Atherosclerosis is linked to cholesterol levels, with patients showing increased IL‐17 levels [84, 85]. The role of IL‐17 in atherosclerosis is debated as some research indicates it may worsen the condition by damaging vascular endothelial cells, triggering inflammatory responses, and increasing lipid accumulation (Figure 3). In a high‐cholesterol diet mouse model, IL‐17 was found to worsen arterial plaque formation and instability by influencing immune cell infiltration, especially macrophages, into the arterial wall [86]. This process involves the secretion of chemokines like CCL2 and CXCL1, which attract immune cells to the vessel wall and transform them into foam cells, furthering lipid accumulation. Conversely, IL‐17‐deficient mice exhibited a notable decrease in macrophage marker (F4/80)‐positive cells in the arterial wall, suggesting that IL‐17 deficiency significantly reduces immune cell filtration [87, 88]. In addition, IL‐17 has been implicated in the activation of macrophages through the enhancement of TH1 cell activity, potentially leading to sustained local inflammatory responses and plaque rupture, thereby elevating the risk of cardiovascular events [89]. Conversely, IL‐17A has been proposed to exert a stabilizing effect on atherosclerotic plaques (Figure 3) [90]. IL‐17A facilitates collagen synthesis by augmenting the TGF‐*β* signaling pathway, thus contributing to plaque stability and mitigating the risk of plaque rupture [91]. Research indicates that IL‐17A inhibits the ubiquitin‐mediated degradation of ABCA1 in macrophages, enhancing its stability via the activation of the p38 MAPK pathway [92]. ABCA1 plays a crucial role in mediating the transport of phospholipids and free cholesterol from intracellular compartments to apolipoprotein A‐I, a critical step in initiating reverse cholesterol transport [93]. By upregulating ABCA1 expression, IL‐17 promotes reverse cholesterol transport, thereby reducing lipid accumulation in macrophages and diminishing lipid deposition in the vascular wall, a mechanism that may counteract atherogenesis [94]. The involvement of IL‐17 in atherosclerosis appears to be contingent upon various physiological conditions and microenvironmental contexts, necessitating further investigation into its precise mechanisms. Modulating the IL‐17 signaling pathway holds potential for novel therapeutic strategies in atherosclerosis management, particularly in mediating the equilibrium between its proinflammatory and plaque‐stabilizing roles. This approach may represent a significant advancement in the treatment of the disease.

### 1.5. Targeting IL‐17 Represents a Promising Therapeutic Strategy for Treating Tumors Associated With Dysregulated Lipid Metabolism

**Figure 5:**
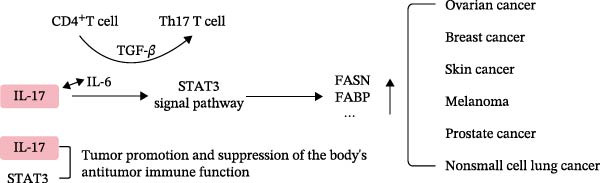
IL‐17 affects lipid metabolism in different malignant tumors through the STAT3 signaling pathway. IL‐17 promotes tumor progression by modulating lipid metabolism through activation of the STAT3 signaling pathway. This process enhances fatty acid uptake, synthesis, and metabolic reprogramming in tumor cells, thereby supporting proliferation, migration, and invasion.

In recent years, the reprogramming of lipid metabolism within tumor cells has been identified as a critical factor in tumorigenesis and progression. Lipids contribute not only as essential components for cell membrane synthesis but also as an important energy source, supporting the rapid proliferation of tumor cells [95, 96]. The upregulation of enzymes related to lipid metabolism, such as ATP citrate lyase (ACLY), ACC, and FASN, is frequently associated with increased tumor aggressiveness and poor prognosis [97–99]. Given the critical role of IL‐17 in lipid metabolism, as previously discussed, targeting IL‐17 may offer a novel therapeutic strategy for cancer treatment. Studies have shown that IL‐17 is markedly overexpressed in a range of solid tumors, such as gastric, colorectal, and HCC, with its expression levels being significantly correlated with tumor aggressiveness [100–105]. IL‐17 has been shown to suppress autophagy and apoptosis in HCC by activating the TAB2/p38 MAPK and IL‐6/STAT3 signaling pathways [106–108]. Additionally, IL‐17 activates the NF‐*κ*B/NOX1 pathway, facilitates the G1‐S phase transition of the cell cycle, and promotes cell proliferation and oxidative stress in gastric cancer [109]. The IL‐17 signaling pathway is mediated by the ACT1‐TRAF2‐TRAF5 complex, which stabilizes target gene expression and modulates the ACT1‐TRAF4‐ERK5 axis to foster tumor cell proliferation [110]. Additionally, IL‐17 contributes to angiogenesis within the tumor microenvironment, thereby indirectly promoting tumor growth by augmenting the nutrient supply to tumor cells [111]. Research has demonstrated that IL‐17 plays a crucial role in regulating lipid metabolism, thereby promoting the development of HCC [69]. Specifically, IL‐17 induces the differentiation of liver progenitor cells (LPCs) into HCC cells that exhibit characteristics of cancer stem cells (CSCs) [112]. The use of IL‐12/IL‐23 antibodies to inhibit IL‐17A/Th17 cells has been shown to significantly impede HCC progression in alcohol‐fed murine models, indicating a contributory role of IL‐17 in HCC pathogenesis. Furthermore, IL‐17A suppresses the caspase‐2‐SREBP1/2 pathway in steatohepatocytes, thereby modulating cholesterol synthesis and enhancing proinflammatory responses, which collectively lead to a reduction in steatosis, fibrosis, and HCC progression. These findings suggest that IL‐17 may facilitate HCC development through its influence on lipid metabolism. Collectively, IL‐17 acts as a critical molecular bridge linking chronic inflammation to lipid metabolic dysregulation across obesity, NAFLD, and HCC, as summarized in Figure 5.

Several studies have elucidated the pivotal role of the IL‐17/STAT3 axis in modulating lipid uptake, synthesis, and catabolism within tumor cells, thereby facilitating tumor proliferation, migration, and invasion. IL‐17 facilitates the activation of STAT3 in tumor cells through IL‐6, which, in conjunction with TGF‐*β*, promotes Th17 differentiation and sustains the Th17 response [108]. Additionally, IL‐17 induces IL‐6 production via NF‐*κ*B activation, establishing a positive feedback loop that enhances the Th17 response. STAT3, in turn, directly binds to the promoters of IL‐17A and IL‐17F, thereby contributing to tumor cell survival, angiogenesis, and immune evasion [113]. Moreover, the activation of STAT3 triggers the SCAP‐SREBP‐1 signaling pathway, leading to the upregulation of fatty acid synthesis, which in turn promotes tumor growth [114]. This activation also increases the expression of lipid metabolism enzymes, such as FASN and fatty acid binding protein (FABP) [114, 115]. The increased expression of these enzymes in tumor cells promotes lipid synthesis, providing the necessary energy and cell membrane components for tumor cell growth, thereby supporting tumor proliferation.

Recent studies have elucidated that IL‐17A facilitates the uptake of fatty acids in ovarian cancer (OvCa) cells via the IL‐17A/IL‐17RA/p‐STAT3/FABP4 signaling axis rather than through the CD36 pathway [116]. Experimental evidence from IL‐17A‐deficient mice, which developed fewer and smaller ovarian tumor nodules compared to their wild‐type counterparts, underscores the pivotal role of IL‐17A in tumor proliferation and metastasis. Research has demonstrated that cancer‐associated adipocytes (CAAs) release fatty acids through lipolysis, which are subsequently transferred to OvCa cells via FABP4, thereby facilitating tumor progression [117]. Clinical investigations have consistently reported elevated levels of IL‐17A expression in advanced and metastatic OvCa cases [118–120]. In the context of breast cancer, STAT3 has been shown to upregulate the transcription of CPT1‐M, a crucial enzyme in fatty acid oxidation (FAO), thereby enhancing lipid metabolism in CSCs and chemoresistant cells [121, 122]. Furthermore, in a DMBA/TPA‐induced skin cancer model, IL‐17‐deficient mice exhibited reduced tumor growth and metastasis, accompanied by decreased levels of IL‐6 and STAT3 phosphorylation, highlighting the critical role of IL‐17 in tumor progression via the IL‐6/STAT3 pathway [123]. In xenograft models of melanoma and bladder cancer, deficiencies in IL‐17 or IL‐17R were observed to diminish proinflammatory and protumorigenic factors, including VEGF and MMPs, thereby limiting tumor growth and reducing both STAT3 activation and immune evasion [124]. IL‐17 facilitates the migration and invasion of nonsmall cell lung cancer (NSCLC) cells through binding to IL‐17RA, a mechanism reliant on the activation of p300 and STAT3 [125]. The p300 protein enhances the transcriptional activity of STAT3 via acetylation at lysine 631, which in turn promotes the expression of MMP19, thereby exacerbating cellular migration and invasion. In vivo studies further corroborated the involvement of p300, STAT3, and MMP19 in the metastasis of NSCLC [114, 115].

## 2. Conclusion

The IL‐17 signaling pathway plays a crucial role in the regulation of lipid metabolism, significantly contributing to the maintenance of lipid homeostasis through its modulation of fatty acid and cholesterol metabolism, adipocyte differentiation, and inflammatory mediators. These findings offer novel insights into the complex interplay between inflammation and metabolic processes. Furthermore, the hyperactivation of the IL‐17 signaling pathway can exacerbate the severity of conditions such as obesity, metabolic syndrome, atherosclerosis, NAFLD, and various malignancies. Consequently, the development of novel pharmacological agents or cotargeted therapeutic strategies aimed at the IL‐17 signaling pathway may provide innovative treatment options for diseases associated with dysregulated lipid metabolism.

## Funding

No funding was received for this study.

## Conflicts of Interest

The authors declare no conflicts of interest.

## Data Availability

Data sharing is not applicable to this article as no datasets were generated or analyzed during the current study.
